# Assessment of Anxiety and Local Anesthesia Experiences in Dental Students Using the Modified Dental Anxiety Scale (MDAS)

**DOI:** 10.3390/dj13100445

**Published:** 2025-09-28

**Authors:** Emilia Bologa, Andra Claudia Tărăboanță-Gamen, Ionuț Tărăboanță, Otilia Boișteanu, Alexandra-Lorina Platon, Șerban-Ovidiu Stelea, Ana-Maria Andreea Simionescu, Anca Irina Grădinariu, Alina Jehac, Cristina Bologa, Carmen Gabriela Stelea

**Affiliations:** 1 Faculty of Dental Medicine, Grigore T. Popa University of Medicine and Pharmacy, 16 Universitatii Str., 700115 Iasi, Romania; bologa.emilia@umfiasi.ro (E.B.); ionut-taraboanta@umfiasi.ro (I.T.); otilia.boisteanu@umfiasi.ro (O.B.); alexandra.lorina.platon@umfiasi.ro (A.-L.P.); stelea_serban-ovidiu@d.umfiasi.ro (Ș.-O.S.); anamaria.simionescu@umfiasi.ro (A.-M.A.S.); anca-gradinariu@umfiasi.ro (A.I.G.); alina.jehac@umfiasi.ro (A.J.); carmen.stelea@umfiasi.ro (C.G.S.); 2 Faculty of Medicine, Grigore T. Popa University of Medicine and Pharmacy, 16 Universitatii Str., 700115 Iasi, Romania; cristina.bologa@umfiasi.ro

**Keywords:** dental anxiety, Modified Dental Anxiety Scale (MDAS), dental students, local anesthesia

## Abstract

**Background and Objectives**: Dental anxiety can hinder both treatment delivery and dental education. Few studies have examined this issue among Romanian dental students. This study assessed the prevalence, intensity, and main procedural triggers of dental anxiety, with a focus on experiences related to local anesthesia. **Methods**: A cross-sectional survey was conducted (January–May 2024) among 122 fourth-year students using the validated Romanian version of the Modified Dental Anxiety Scale (MDAS) and additional items on demographics, prior anesthesia and perceived complications. Data analysis included descriptive statistics, chi-square tests, and *t*-tests. **Results**: Overall, 21.3% of respondents scored in the low anxiety range, 75.4% in the moderate range, and 3.3% in the severe range (MDAS ≥ 19). No significant gender differences were identified (*p* > 0.05). Injections with local anesthetic were rated as the most distressing procedure, followed by drilling, whereas scaling was reported as least stressful. The majority (86.9%) had undergone previous local anesthesia, with very few adverse events recalled. **Conclusions**: Romanian dental students reported moderate dental anxiety overall, with local anesthesia injections as the main trigger. The lack of gender differences points to a potential buffering effect of clinical exposure. Incorporating structured anxiety management into dental curricula may enhance both student well-being and patient care.

## 1. Introduction

Dental anxiety represents one of the most persistent barriers to effective oral health care. Despite major technological and procedural advances in dentistry, prevalence rates have remained stable since the 1960s. Between 10% and 20% of adults experience clinically significant dental anxiety, while 3–5% meet criteria for severe phobia [1,2]. Higher levels are often reported in younger individuals and pregnant women [3,4,5]. These figures confirm the substantial global burden of dental anxiety and underline its significance as both a clinical and public health issue. The World Health Organization recognizes odontophobia as a specific condition, given its association with treatment avoidance, deterioration of oral health, and impaired psychosocial functioning [2].

The origins of dental anxiety are multifactorial [6]. In addition, a recent study highlighted that sensory sensitivity and pain catastrophizing function as independent predictors of dental anxiety, with alexithymia exerting indirect effects through these mechanisms [7]. Painful experiences during childhood are well-recognized triggers, but indirect pathways such as observing anxious relatives or negative cultural messages about dentistry also play an important role [8]. More recently, cognitive models have emphasized that perceptions of unpredictability and lack of control are central drivers of fear, sometimes more influential than past experiences themselves [9]. Socio-demographic variables, including female gender, younger age, and psychiatric comorbidities, further contribute to individual vulnerability [10,11].

The consequences extend far beyond momentary discomfort. Dental anxiety leads to avoidance of care, progression of untreated disease, and complex restorative needs, perpetuating a vicious cycle of fear and deteriorating oral health [12]. It is also closely linked with diminished oral health–related quality of life, affecting daily functioning, psychological well-being, and social interaction [13,14]. Dental anxiety and negative dental experiences, both in childhood and adulthood, were significantly associated with poorer attitudes towards oral hygiene and reduced self-efficacy in maintaining oral health [15]. In clinical settings, anxiety complicates behavior management and may increase reliance on sedation or general anesthesia [16].

Reliable measurement tools are crucial for identifying and comparing anxiety levels. While early instruments such as Corah’s Dental Anxiety Scale and the Dental Fear Survey provided important foundations, the Modified Dental Anxiety Scale (MDAS) has become the most widely adopted, particularly due to its brevity and its inclusion of an item specifically addressing fear of local anesthesia injections [17,18,19]. The MDAS has been validated internationally, including in Romania and consistently demonstrates excellent reliability [20]. More comprehensive tools such as the Index of Dental Anxiety and Fear (IDAF-4C+) have also shown strong psychometric properties, though their greater length may limit feasibility in educational settings [21,22].

Dental students represent a particularly relevant population for investigation, as their clinical training is expected to reduce anxiety. However, existing research indicates that they continue to experience considerable levels of fear, particularly in relation to procedures involving local anesthesia injections and dental drilling [23,24]. An umbrella review suggested that almost one third of university students overall report clinically significant anxiety, and dental students must manage both their personal anxieties and the task of caring for anxious patients [25]. Encouragingly, their confidence in managing such patients improves throughout training [26].

However, no study to date has examined dental anxiety and experiences with local anesthesia in Romanian dental students. This study therefore aimed to assess anxiety levels and procedure-specific triggers in a cohort of fourth-year students at the Faculty of Dental Medicine, “Grigore T. Popa” University of Medicine and Pharmacy, Iași, using the validated Romanian version of the Modified Dental Anxiety Scale (MDAS). The findings are expected to support the integration of structured anxiety management strategies into dental curricula and to inform approaches for improving both student well-being and patient care. In this way, the study complements international evidence by providing novel data from an underrepresented European population. The null hypothesis tested in this study stated that there are no statistically significant differences in dental anxiety levels among students based on gender, previous experiences with local anesthesia, or specific dental procedures.

## 2. Materials and Methods

### 2.1. Study Design and Participants

This cross-sectional study was conducted between January and May 2024 on fourth-year dental students from the Faculty of Dental Medicine at “Grigore T. Popa” University of Medicine and Pharmacy, Iași, Romania. Participation was voluntary, and informed consent was obtained from all students prior to enrollment. The study protocol was approved by the University Ethics Committee (approval no. 368, on 8 December 2023). The inclusion criteria required participants to be enrolled in the fourth year of dental school. Exclusion criteria included a history of psychiatric illness, refusal to participate, or incomplete questionnaire submission.

The sample size (n = 122) was determined to be adequate based on an a priori power analysis performed using G*Power software (version 3.1.9.7; Heinrich Heine University Düsseldorf, Germany). Assuming a medium effect size (Cohen’s d = 0.5), a significance level of α = 0.05, and a statistical power of 80%, the analysis indicated that a minimum of 102 participants would be required. Therefore, the final sample of 122 students provided sufficient power to detect clinically meaningful differences between subgroups.

### 2.2. Data Acquisition

The instrument used was the Modified Dental Anxiety Scale (MDAS), which consists of five items scored on a five-point Likert scale, with total scores ranging from 5 to 25. The Romanian version of the MDAS has previously been validated and demonstrated excellent psychometric properties [20]. At the methodological level, the inclusion of an item specifically addressing the local anesthetic injection has been emphasized as a distinctive strength of the MDAS, which has been conceptualized along two latent constructs, anticipatory and treatment-related dental anxiety, with the injection item playing a central role in capturing sensitivity to invasive stimuli [27]. Additional items collected demographic data (age, gender) and information about previous experiences with local anesthesia, perceived complications, and procedures considered most anxiety-inducing.

Scores ≥19 indicate high dental anxiety or dental phobia. Participants were also asked whether they had previously received local anesthesia, what type of anesthesia they found most anxiety-inducing (seven possible techniques were listed), and whether they had experienced any complications, such as pain, hematoma, paresthesia, trismus, or infection.

Data was collected using a structured, anonymous questionnaire distributed in print during regular teaching sessions. The questionnaires were self-administered under supervision in class, ensuring standardized completion. All questionnaires were fully completed, so no missing data were present. Completed questionnaires were coded and entered into a database for analysis.

### 2.3. Statistical Analysis

Descriptive statistics (means, standard deviations, frequencies, and percentages) were calculated for all demographic variables and Modified Dental Anxiety Scale (MDAS) scores.

Assumptions of normality (Shapiro–Wilk, *p* > 0.05) and homogeneity of variances (Levene’s test, *p* > 0.05) were verified prior to statistical analysis. Effect sizes were reported to complement statistical significance, including Cohen’s d (≥0.20 for small, ≥0.50 for medium, ≥0.80 for large effects) for group comparisons and odds ratios (OR) with 95% confidence intervals.

Group comparisons were conducted using independent samples *t*-tests for continuous variables and chi-square tests for categorical variables. All tests were two-tailed, and a *p*-value < 0.05 was considered statistically significant.

## 3. Results

A total of 122 fourth-year dental students participated in the study (79 females, 43 males). The mean age was 22.94 ± 2.26 years for females and 23.14 ± 2.82 years for males, with no significant difference between groups (*p* > 0.05).

The age difference between female (22.94 ± 2.26 years; *n* = 79) and male participants (23.14 ± 2.82 years; *n* = 43) was negligible (Hedges’ g = −0.08). Prior experience with local anesthesia was reported more frequently by women (92.4%) than by men (76.7%), corresponding to an odds ratio of 3.69 (95% CI [1.24, 10.99]; φ = 0.22). For the individual MDAS items, the strength of the association between gender and response was consistently small, with Cramér’s V ranging from 0.09 (Q1) to 0.21 (Q3), aligning with the nonsignificant χ^2^ tests.

Analysis of the MDAS revealed that the majority of students presented moderate levels of dental anxiety. The distribution of total scores showed that 26 students (21.3%) reported low anxiety, 50 (41.0%) moderate anxiety, 34 (27.9%) higher moderate anxiety, and 8 (6.6%) high anxiety, while only 4 (3.3%) exceeded the threshold of 19 points, corresponding to very high anxiety. Both genders displayed similar profiles, and *t*-test analysis confirmed the absence of statistically significant differences between male and female students (*p* > 0.05) (Table 1).

**Figure 1:**
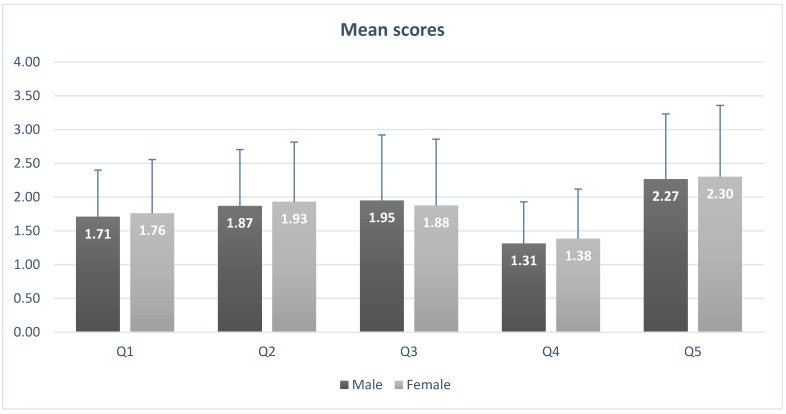
Mean MDAS item scores with standard deviations for male and female students.

As is observed in Figure 1, the analysis of individual items revealed distinct patterns. For the prospect of visiting the dentist the following day (Q1), the mean score was 1.77 ± 0.84; most students reported little or no anxiety, with 38.5% selecting “not anxious” and 50.8% “slightly anxious,” while extreme anxiety was recorded in only 1.6% of cases. Sitting in the waiting room (Q2) elicited similar results, with a mean score of 1.89 ± 0.92; over half of respondents (52.5%) reported slight anxiety, while 28.7% were not anxious at all. Drilling (Q3) provoked higher levels of anxiety, with a mean score of 2.05 ± 1.04; 43.4% of students were slightly anxious, 12.3% fairly anxious, and 4.1% either very or extremely anxious. Scaling and cleaning (Q4) were perceived as the least stressful, with a mean score of 1.38 ± 0.73; nearly two-thirds of respondents (65.6%) reported no anxiety. Local anesthesia injections (Q5) emerged as the strongest trigger, with a mean score of 2.29 ± 1.02; although 21.3% reported no anxiety, 41.0% indicated slight anxiety, 27.9% fairly anxious, and almost 10% (9.9%) were very or extremely anxious. Comparative analysis with the Chi-square test showed no significant gender differences for any of the five items (*p* > 0.05).

Regarding previous experiences with anesthesia (Q6), 106 students (86.9%) reported having received local anesthesia at least once, compared with 16 students (13.1%) who had not; this difference was statistically significant (*p* < 0.001) (Table 2).

**Figure 2:**
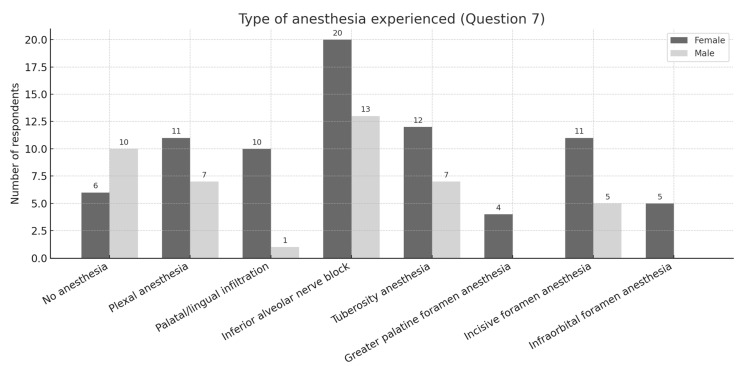
Distribution of responses to question 7 regarding the type of anesthesia experienced, by gender.

Further analysis of the type of anesthesia (Q7) revealed that female students most frequently reported inferior alveolar nerve block, while male students most often indicated tuberosity block; in both groups, greater palatine block was rarely or never chosen (Figure 2).

**Figure 3:**
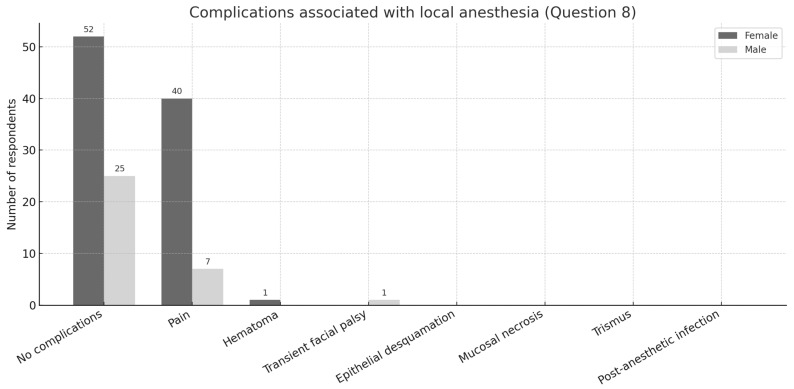
Distribution of responses to question 8 on complications associated with local anesthesia, by gender.

In terms of complications (Q8), the large majority reported no adverse effects (52 females and 25 males). A minority indicated transient discomfort, whereas none reported serious or persistent complications (Figure 3).

## 4. Discussion

The present study assessed dental anxiety among Romanian dental students using the validated Romanian version of the MDAS and found that most participants experienced moderate levels of fear. Only a small proportion of students reached the threshold for higher levels of anxiety, and no extreme phobia was observed. Our findings are consistent with data from several European countries, which reported moderate levels of dental anxiety among dental students. By integrating these comparisons, it becomes clear that the anxiety patterns observed in our cohort align with those documented in other European populations [3,10,28,29].

Local anesthesia injections were identified as the most anxiety-inducing procedure, whereas scaling was consistently perceived as the least stressful. These results expand the limited evidence on Romanian student populations and highlight the relevance of addressing procedure-specific fear in educational settings.

Epidemiological research has consistently demonstrated that dental anxiety is a widespread condition, with prevalence rates of 10–20% for clinically significant fear and 3–5% for severe phobia [1,2]. In some populations, such as Turkish young adults, prevalence may exceed 30% [3,10]. The comparatively lower rates of severe anxiety in our cohort suggest that professional training contributes to reducing generalized fear, though procedure-specific anxieties may persist. Similar findings have been reported in India and Malaysia, where injections and drilling remained the main sources of fear throughout training, and in Portugal, where first-year students demonstrated significantly higher anxiety levels than fifth-year students, particularly in relation to surgical and endodontic procedures [23,24,30]. These findings underscore the protective effect of progressive clinical exposure while emphasizing the persistence of procedural triggers such as injections.

A recent study from Pakistan identified significantly higher levels of dental anxiety among women compared with men in a large adult cohort, with 75% of female participants presenting MDAS scores above 10 and 20% meeting the criteria for dental phobia [31]. Unlike our sample of dental students, their research targeted the general population, which may explain the observed gender disparities. This contrast supports our interpretation that medical education helps equalize emotional responses, attenuating gender-related differences in dental anxiety.

Comparisons with other academic cohorts support this interpretation. Globally, around one third of university students suffer from clinically relevant anxiety disorders [25]. Dental students are therefore not immune to broader psychological vulnerabilities. In fact, the coexistence of personal anxiety and the responsibility of managing anxious patients creates a dual challenge. Surveys confirm that students are aware of the importance of anxiety management and that their competence improves as training progresses [26]. However, the persistence of fear regarding anesthesia injections in our cohort suggests that technical competence is insufficient unless paired with emotional coping strategies. The persistence of injection-related anxiety despite repeated exposure and the absence of complications highlights the anticipatory and physiological nature of fear. Recent studies have shown that local anesthesia can elicit measurable hemodynamic changes, including increases in blood pressure and heart rate, even without adverse outcomes [32,33]. This aligns with our observation that students consistently perceived anesthesia as a highly stressful experience.

Innovative interventions, such as distraction through virtual reality during anesthesia, have been tested to reduce anticipatory fear and improve patient comfort [34]. The fact that anesthesia emerged as the dominant trigger in our study supports the rationale for implementing such targeted approaches in both education and practice.

Dental anxiety carries important consequences for oral health and psychosocial well-being. High levels of fear are associated with irregular dental attendance, higher caries experience, and worse quality of life [13]. Recent findings from a large German cohort further confirmed that individuals with elevated dental anxiety scores reported poorer oral-health-related quality of life and lower engagement in preventive behaviors such as calculus removal and professional teeth cleaning [29]. Even in our student cohort, which had regular access to care, the persistence of moderate anxiety indicates that anticipatory distress alone can influence behaviors and expectations. This resonates with the description of a vicious cycle in which fear leads to avoidance, deterioration of oral health, and further anxiety, reinforcing avoidance in the long term [12]. The presence of this mechanism, even in dental students, illustrates the robustness of the cycle. The choice of assessment instrument is another aspect worth discussing.

Our study employed the Modified Dental Anxiety Scale, which is concise and widely validated. By including a specific item on local anesthesia injections, the MDAS demonstrates particular sensitivity to procedure-related fear, making it highly relevant for academic populations. The instrument has been validated in Romania with excellent reliability and is considered practical for large-scale studies [20]. Alternative measures such as Corah’s Dental Anxiety Scale or the Dental Fear Survey are longer and capture broader constructs, while the Index of Dental Anxiety and Fear offers a multidimensional profile including emotional, cognitive, behavioral, and physiological components [17,21,22]. Nevertheless, for academic populations, the brevity and specificity of the MDAS make it particularly suitable, and our findings further confirm its sensitivity to procedure-related triggers. The robustness of the MDAS has also been confirmed in populations beyond adults. A validation conducted among Finnish schoolchildren aged 9–12 years demonstrated strong internal consistency and significant correlations with other pediatric dental fear measures [35]. This further reinforces the psychometric soundness and cross-context applicability of the MDAS as a brief yet reliable measure.

The literature also highlights the multifactorial origins of dental anxiety. Direct conditioning through painful or traumatic experiences during childhood has been recognized as a key factor [8]. However, vicarious learning through observation of parents or peers, as well as negative cultural narratives about dentistry, also contribute to the development of fear. Cognitive models propose that expectations of pain and perceived lack of control are central mechanisms driving anxiety [9]. Our findings illustrate that anticipatory mechanisms and subjective perceptions can outweigh actual clinical experiences. Further, recent theoretical contributions have expanded the understanding of dental fear as part of a broader continuum of psychological vulnerability. Dental anxiety has been conceptualized as linked with general anxiety sensitivity and sensory over-responsivity, phenomena that persist across the lifespan [16]. This interpretation aligns with our data, where advanced students continued to display moderate anxiety despite extensive exposure to dental procedures.

The persistence of such responses underlines the need for educational strategies that target resilience, not only technical mastery. From an educational perspective, our findings emphasize the importance of embedding anxiety management into the curriculum. Students must learn to control their own fears and simultaneously acquire the competence to support anxious patients. This requires a dual approach: providing opportunities for reflection on personal experiences of anxiety and training in behavioral and communicative techniques to manage patient fear. Evidence indicates that structured training in this area improves both self-confidence and clinical performance [26]. A particular focus should be placed on procedure-related fears, ensuring that students develop both technical competence and coping skills for managing stressful interventions. The impact of dental anxiety on students’ professional development must not be underestimated. Practitioners who retain personal anxieties may encounter difficulties in administering procedures such as anesthesia with confidence, potentially affecting patient trust and treatment outcomes. Conversely, students who overcome their fears may be better positioned to empathize with anxious patients. Addressing these issues within education thus has benefits both for students’ well-being and for future patient care. Faculty could also integrate short workshops on anxiety management into clinical training to provide students with practical coping strategies. Similar approaches have been shown to reduce anxiety in patients; for example, in a Saudi study demonstrated that showing a short educational video before tooth extraction significantly decreased fear and anxiety compared with verbal explanation alone. Although this study involved patients rather than students, it highlights the importance of structured communication and educational interventions for managing dental anxiety [36].

Limitations of the present study should be acknowledged. The sample was restricted to a single institution, which may limit generalizability. The cross-sectional design provides only a snapshot and cannot assess changes across the years of study. Although the MDAS is reliable and widely validated, it is limited to five items and does not capture all dimensions of anxiety. Future research should adopt longitudinal and multicenter designs, incorporating additional instruments such as the Dental Fear Survey or the IDAF-4C+ [17,21,22]. Intervention studies would also be valuable, testing approaches such as virtual reality distraction or simulation-based training, with the aim of reducing both personal and patient anxiety [34].

Despite these limitations, the present study contributes valuable evidence by documenting that moderate dental anxiety persists among Romanian dental students, that gender differences are attenuated in this academic context, and that anesthesia injections remain the most salient trigger of fear. These findings align with international data while also offering unique insights into the Romanian context, where empirical research on dental anxiety remains limited. By integrating these results with existing models and cross-cultural evidence, the study highlights the need for comprehensive educational strategies that combine technical competence with psychological support.

## 5. Conclusions

1. The majority of dental students exhibit moderate levels of dental anxiety despite extensive exposure to local anesthesia, suggesting that the fear is largely anticipatory rather than based on adverse clinical outcomes.
2. Local anesthetic injections represent the primary trigger of dental anxiety, surpassing other procedures such as scaling and drilling in terms of perceived distress.
3. No statistically significant gender differences were identified regarding dental anxiety levels, implying that educational exposure may play a balancing role in emotional vulnerability.
4. The Modified Dental Anxiety Scale (MDAS) can be recommended as a practical and validated tool for evaluating dental anxiety in academic populations. However, its limitations, including reliance on self-reported data and restriction to five items, should be acknowledged when interpreting results.
5. The integration of structured anxiety management strategies into the dental curriculum is essential to enhance students’ well-being and improve the quality of patient care.

## Figures and Tables

**Table 1:** Number of respondents to questions 1–5 with Pearson Chi-square values and *p*-values.

Question no.	Gender	Not Anxious		Slightly Anxious		Fairly Anxious		Very Anxious		Extremely Anxious	
		*n*	%	*n*	%	*n*	%	*n*	%	*n*	%
1	Female	30	24.6	42	34.4	6	4.9	0	0	1	0.8
* 0.953	Male	17	13.9	20	16.4	5	4.1	0	0	1	0.8
** 0.813	Total	47	38.5	62	50.8	11	9	0	0	2	1.6
2	Female	21	17.2	45	36.9	11	9	1	0.8	1	0.8
* 2.806	Male	14	11.5	19	15.6	7	5.7	2	1.6	1	0.8
** 0.591	Total	35	28.7	64	52.5	18	14.8	3	2.5	2	1.6
3	Female	31	25.4	39	32	7	5.7	1	0.8	1	0.8
* 5.497	Male	18	14.8	14	11.5	8	6.6	1	0.8	2	1.6
** 0.240	Total	49	40.2	53	43.4	15	12.3	2	1.6	3	2.5
4	Female	48	39.3	24	19.7	4	3.3	2	1.6	1	0.8
* 4.951	Male	32	26.2	6	4.9	3	2.5	2	1.6	0	0
** 0.292	Total	80	65.6	30	24.6	7	5.7	4	3.3	1	0.8
5	Female	17	13.9	32	26.2	24	19.7	4	3.3	2	1.6
* 1.669	Male	9	7.4	18	14.8	10	8.2	4	3.3	2	1.6
** 0.796	Total	26	21.3	50	41	34	27.9	8	6.6	4	3.3

* Pearson Chi Value; ** *p* Value.

**Table 2:** Distribution of responses to question 6 (Q6) by gender, with chi-square test results.

		Question 6		Total	
		No	Yes		*p* Value (*t*-Test)
Gender	Female	6	73	79	*p* < 0.001
	Male	10	33	43	
Total		16	106	122	

## Data Availability

The original contributions presented in this study are included in the article. Further inquiries can be directed to the corresponding author.

## Abbreviations

The following abbreviations are used in this manuscript:

MDAS	Modified Dental Anxiety Scale
DAS	Dental Anxiety Scale
IDAF-4C+	Index of Dental Anxiety and Fear
WHO	World Health Organization
SD	Standard Deviation
CI	Confidence Interval
